# The Effect of Exercise on Pulsatility Index of Uterine Arteries and Pain in Primary Dysmenorrhea

**DOI:** 10.3390/jcm12227021

**Published:** 2023-11-10

**Authors:** Zoltán Kovács, Gabriella Hegyi, Henrik Szőke

**Affiliations:** 1 Department of Obstetrics, Robert Hospital, 1135 Budapest, Hungary; 2 Doctoral School of Health Sciences, Faculty of Health Sciences, University of Pécs, 7621 Pécs, Hungary; gabriella.hegyi@etk.pte.hu (G.H.); henrik.szoke@etk.pte.hu (H.S.); 3 Department of Integrative Medicine, Faculty of Health Sciences, University of Pécs, 7621 Pécs, Hungary

**Keywords:** primary dysmenorrhea, pain, exercise, pulsatility index, uterine artery

## Abstract

Background: Primary dysmenorrhea (PD) is one of the most common diseases in women of reproductive age. Our aim was to examine whether a twice-weekly thirty-minute exercise intervention could result in a difference in the pulsatility index (PI) of the uterine arteries (UAs) and level of menstrual pain in patients with PD. Methods: In our prospective observational trial, the observation period included one spontaneous menstrual cycle and the consecutive time of the next menstruation of all participants, aged 18–44, with no extensive sports experience. In total, 73 volunteers were enrolled: 38 persons in the intervention group (IG) and 35 in the control group (CG). The intervention program was accompanied by music, performed in groups under the supervision of a qualified instructor in Hungary. The primary outcome was the difference between the IG and CG regarding the PI values of UAs at the 1st and the 2nd ultrasound (US) Doppler flowmetry. The secondary outcome was the difference between the IG and CG regarding the PI of UAs and menstrual pain measured by using the Numeric Rating Scale and adherence to the intervention. Statistical tests such as an independent-samples *t*-test, chi-square test, Mann–Whitney test and analysis of covariance (ANCOVA) were used during the analyses. Results: Examining the mean of the PI of UAs in the IG and the CG at the 1st and the 2nd US measurement, a significant difference was found in the change in the measured value (Z = −2.545; *p* = 0.011). The IG showed a significantly higher increase in the mean of the PI of UAs (Median = 0.825) than the CG (Median = 0.130). The difference in the PI of the UAs of the IG and the CG is not related to the level of pain in any group (*p* = 0.336) and not related to the whole sample (*p* = 0.354); furthermore, the level of pain did not significantly differ between the two groups. Conclusions: Our study is the first to document the significant effects of mild-to-moderate exercise training on the change in the PI of the UAs in individuals with PD. The IG had a reduced blood flow due to circulatory redistribution after exercise. The level of menstrual pain of primary dysmenorrhea patients is independent of the level of blood circulation regarding the PI of the UAs. Randomized controlled studies with more participants and a longer research period are needed to confirm our findings regarding the association between regular exercise and the PI of UAs. The study was registered at clinicaltrials.gov: NCT04618172.

## 1. Introduction

Primary dysmenorrhoea (PD) is a painful condition that occurs monthly with menses and has no clear underlying disease. Epidemiological studies have shown that this disorder is one of the most common issues among both adolescent girls and adult women of reproductive age [1]. PD characteristically begins within three years of menarche [2]. In contrast to PD, secondary dysmenorrhoea is associated with underlying pathology, including endometriosis, fibroid or pelvic inflammatory disease (PID) [3].

Hungarian data on PD prevalence among Hungarian women of childbearing age are very limited. In a Hungarian epidemiological study published in 2005, 1852 of the 2337 adolescent girls who responded to the questionnaire survey reported painful menstruation (79.2%). In total, 30% of girls with dysmenorrhoea described their symptoms as mild, while 67% of them reported it as very strong and unpleasant [4]. In a systematic review and meta-analysis of studies including over 21,000 young women from 38 different countries, the prevalence of dysmenorrhea was 71.1% [5]. This study demonstrated the significant academic impact of dysmenorrhea, with 20.1% reporting absence from school or university due to dysmenorrhea and 40.9% reporting classroom performance or concentration being negatively affected [5]. Severe symptoms of PD are estimated to affect 10 to 25% of menstruating women worldwide [6,7], resulting in 10% of women being unable to work [8]. PD has a profound negative impact on an individual’s quality of life, through social, physical, psychological and emotional impairment [8,9]. 

Rencz et al. completed a Hungarian study examining the health-related quality of life values for severe and mild PD using 10-year time trade-off and willingness-to-pay methods [6]. They studied 1836 participants who met the criteria for PD, originally recruited from a 2015 national convenience sample of internet survey responders. In total, 70% of participants who experienced dysmenorrhea reported an impact on their work/study and social activities, with 16% reporting extreme impact on these domains. Quality-adjusted life year loss was comparable to chronic migraine, asthma and type 1 diabetes.

Given the connections between dysmenorrhea and other chronic pain conditions, treating dysmenorrhea early and effectively is more important than previously recognized [10]. Common medical dysmenorrhea treatments vary in their effectiveness across individuals. A significant percentage of women reported the ineffectiveness of medical dysmenorrhea treatments [10]. Women appear to be actively looking for and using non-pharmacological lifestyle interventions, e.g., exercises to relieve menstrual symptoms [11,12,13]. In our study, we used the American College of Sports Medicine definition of exercise as “physical activity characterized by using planned and structured repetitive movements to increase or maintain physical fitness” [14,15]. This also incorporates mild- and moderate-intensity exercise. Systematic reviews and meta-analyses also recommend exercise as a self-care strategy and non-pharmacological therapy for PD, in addition to its other numerous health benefits [16,17]. A Canadian national guideline recommends regular exercise as a non-pharmacological alternative therapy for PD [18]. Some previous randomized control trials have shown that regular exercise three times a week [19], or even twice a week, reduced pain in PD [20].

The UAs provide most of the perfusion of the uterus; therefore, assessment of their vascular properties is expected to provide important information on the uterine blood supply and its ability to fulfil its essential role in human reproduction. The non-invasive and easy-to-use Doppler ultrasound equipment has become a mainstay in the assessment of such properties [21,22,23]. Using Doppler ultrasound, different impedance parameters can be estimated. Of these, PI has been given more importance as it seems to more accurately describe the blood velocity waveform. The reference ranges for the PI values of UAs during menstrual cycles in healthy patients were examined in different studies [21,22,23]. The relationship between menstrual pain and PI values of UAs during different phases of the menstrual cycle in healthy patients and those with PD was also examined in other studies [8,24,25]. Additionally, there is solid evidence from different studies that the perfusion of the UAs and uterine arterioles is reduced in women with PD in comparison to healthy women [22,24,25,26]. Its potential cause may be the abnormal elevation in the levels of prostaglandins (e.g., PGE2 and PGF2α) and leukotrienes (e.g., leukotrienes C4, D4), as well as changes in the production of nitric oxide in the endometrium and the menstrual fluid, resulting in both myometrial contraction and vasoconstriction of uterine vessels, including uterine arterioles. Both mechanisms of action produce hypoxia and ischemia that lead to pain in patients with PD. PGF2α also lowers the threshold for pain perception by sensitizing the nerve receptors [3,27]. Furthermore, a positive correlation has been shown between endometrial prostaglandin levels and pain severity [8].

To our knowledge, our study is the first in the literature investigating the effects of mild-to-moderate twice-weekly thirty-minute exercise as a non-pharmaceutical lifestyle intervention on the PI values of UAs in individuals with PD. Our hypotheses were: (1) There would be a significant increase in the PI of UAs in the mild-to-moderate exercise intervention group (IG) compared to the control group (CG). (2) There would be a significant difference between the IG and CG regarding the PI of UAs and menstrual pain measured by using the Numeric Rating Scale (NRS).

In this study, we examined one primary and two secondary outcomes. We will publish further results from our complex research based on different questionnaires with more data regarding the IG and the CG (change in physical, psychological and behavioural complaints of premenstrual syndrome measured by Prospective Record of the Impact and Severity of Menstrual Calendar; change in the dysmenorrhea complaints measured by using the NRS; and change in body awareness measured by the Hungarian Body Awareness Questionnaire) in other articles to avoid a too-complicated and difficult-to-understand scientific article. 

## 2. Materials and Methods

### 2.1. Study Design

We performed a prospective observational trial. At the start of the study period, entry criteria included age between 18 and 44 years, body mass index (BMI) between 17 and 35, regular menstrual cycle duration between 21 and 35 days and menstrual duration between 3 and 7 days. All IG patients were new to this exercise series. With respect to the Committee Opinion No. 760 of the American College of Obstetricians and Gynecologists (ACOG) [27], the initial diagnosis for all patients presenting with PD included a medical, family, psychosocial, gynaecological and menstrual history to confirm whether a patient had PD or symptoms characteristic of secondary dysmenorrhea via outpatient gynaecological department. When a patient only presents with symptoms of PD, manual pelvic and vaginal ultrasound examinations are not necessary [27]. Exclusion criteria included pregnancy, taking oral contraceptive pills, painkillers for PD, any case of secondary dysmenorrhoea (e.g., ovarian cysts, uterine malformations, endometrial polyps or endometriosis, PID), regular medication for psychiatric neurological or endocrinological conditions, established professional or regular sports performance, and major traumatic life events (death in the family, etc.) within three months prior to the start of the study. We also excluded patients who became pregnant during the study and those who decided to discontinue participation before the end of the study. We also excluded participants who did not complete the questionnaire correctly for three consecutive days or attend the ultrasound flowmetry measurements. Women were allowed to volunteer to participate after being informed about the study and its requirements.

### 2.2. Study Population

**Figure 1:**
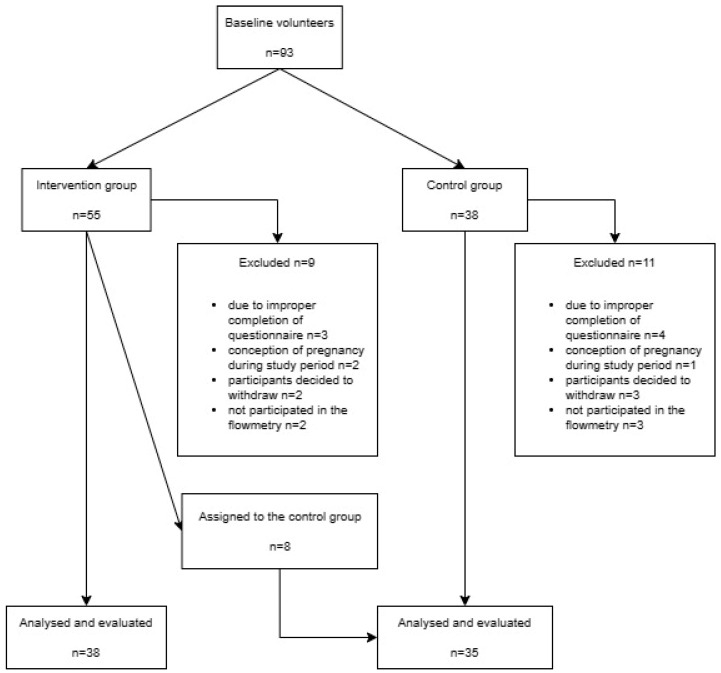
Flow chart of the study.

Study population in the IG and CG can be seen in Figure 1.

In the IG, eight participants reported not performing the exercises twice weekly on a regular basis. These participants were subsequently assigned to the CG.

### 2.3. Sample Size and Assignment

Convenience sampling was used because no preliminary or earlier data were available when examining the effects of exercise on the PI values of UAs in individuals with PD. The IG and CG were non-randomized as participants self-selected under real-world conditions to be included either in the exercise intervention or the control arms of the study. Self-selection included voluntary participation in learning a new set of exercises and willingness to regularly practice in a group afterwards. We also sought to make our research flexible and realistic, with women of all ages and from all parts of the country in the reproductive life stage who volunteered to participate in the research, even travelling from remote locations to practice together. This volunteering was time-consuming, costly and not compatible with work or family commitments for all participants. In view of the above, as well as the ethical considerations, we did not want to deprive volunteers of the opportunity to make an effort to learn practice intervention.

### 2.4. Doppler Ultrasound Flowmetry Assessment

The ultrasound examination was performed with the woman in the supine position and in the morning. Uterine artery Doppler flowmetry assessment was performed using a Voluson E6 (GE Healthcare Technologies, Chicago, IL, USA) ultrasound machine with multi-frequency transvaginal transducers. The evaluations were performed by a single operator with extensive experience in performing a Doppler ultrasound, using a transvaginal transducer to avoid inter-observer variation. After excluding uterine, Fallopian tube and ovarian pathology with the conventional ultrasound, a sagittal image of the uterus was taken, including the cervical canal and the internal cervical canal. The transducer was then gently tilted to the side and a colour flow mapping was used to identify the right and left uterine artery at the level of the internal uterine orifice. Pulsed-wave Doppler was used, with a 2 mm sampling gate being used to image the entire vessel and ensure that the angle of insonation was less than 30°. PI of UAs were measured automatically, as follows:

PI = systolic peak velocity − end diastolic velocity/mean velocity during cardiac cycle.

Three similar successive waveforms were obtained, and the average PI of the left and right UAs was calculated.

To determine the average intraobserver error, a single operator repeated the transvaginal ultrasound examination for five patients each, five consecutive times. The intraobserver variation coefficient was found to be 7–9% for the PI of UAs.

### 2.5. Outcomes

#### 2.5.1. Primary Outcome

The primary outcome was the difference between the IG and the CG regarding the PI of the UAs at the 1st and the 2nd examination, measured by using ultrasound Doppler flowmetry.

#### 2.5.2. Secondary Outcomes

The secondary outcomes were as follows:

(1) The difference between the IG and CG regarding the PI of UAs and menstrual pain measured by using the NRS.
(2) Adherence to the intervention.

### 2.6. Exercise Intervention Program

Upon entry, participants placed in the IG were given 4 h of training to learn the exercise intervention program. These exercises are different from general exercises, e.g., hiking, running or swimming. The intervention program consisted of a total of 19 exercises accompanied by music [28,29,30], performed twice a week in groups under the supervision of a qualified instructor in Budapest, Hungary. The exercises were a carefully structured, intense and methodical series of movement sequences, including a 5 min warm-up exercise at the beginning and 5 min cooling-down exercises at the end of the intervention. The exercise intervention program lasted 30 min. There was no break between the 19 exercises—the participants exercised continuously. 

Most of the exercises were performed in a standing position, with each exercise repeated continuously and successively. These included different side, cross, forward and back steps; moving back and forth, and right–left; tapping; turning the upper body or the whole torso; leaning forward and back with straight legs; leaning forward and back with flexed knees; tilting to the sides; circulatory movements with arms; stretching and rotating the shoulders; shoulders and arms twisting right and left (head and hips facing forward); rising to the balls of the feet and maintaining balance; squatting-down and standing-up movements; swinging the legs forwards and to the sides; tilting the hips to the sides and forward; turning hips to the sides while knees were bent; pulling knees to chest, then kicking and pulling knees up again and alternating legs; bouncing deeply while one arm swings forward and the other arm swings backwards (alternating the arms as one moves); moving right leg forward and bending; bending left knee and tilting pelvis, then stretching out the left knee (right leg stays bent the entire time) and left leg forward (repeating movements on the left side); leaning forward and circling around while stomping out to the side, first with the right leg then with the left leg when stepping out. Some of the exercises were performed in a sitting position, repeating each exercise continuously and successively. These exercises were as follows: stretching legs, pulling back, then bringing legs to the side and lengthening, before switching legs and repeating the exercise; flexing feet and stretching legs forward, then lifting legs 45 degrees, holding this position while counting to 4, pulling knees to the chest, and leaning forward onto straight legs. Some of the exercises were performed in a supine position repeating each exercise continuously and successively: lying on back, stretching legs out toward the ceiling, lowering legs and stopping at 45 degrees to hold, then lowering legs again, stopping 5 cm from the floor and dropping heels with a thud; lying on back, hugging knees with hands to chest and squeezing glutes, then relaxing. Some of the exercises were performed in a prone position, repeating each exercise continuously and successively: lying face-down, squeezing glutes and lifting right leg, holding this for a count of 4, before repeating the exercise with the left leg, and then with both legs together.

The exercises were performed by the participants in the IG twice a week during the study period, regardless of the day and phase of the menstrual cycle. During the study period, women in the IG and CG were asked not to take any medication, including painkillers. CG subjects did not participate in any exercise intervention.

### 2.7. Data Collection

Recruitment and data collection took place continuously during the study period: 1 March 2019–30 June 2020.

All participants were enrolled for a period of one spontaneous menstrual cycle and the consecutive time of the next menstruation. 

All women in the IG and CG were examined via ultrasound Doppler flowmetry once during one spontaneous menstrual cycle. The date of the vaginal ultrasound scan for each participant in the IG and CG depended on the appointment, as well as what suited the participant and the ultrasound operator. Therefore, some of the ultrasound assessments took place in the follicular phase (but not the days of menses), while some of them took place during the ovulation period and some during the luteal phase according to the length of the participants’ cycle and predicted ovulation period. In the IG, each participant took part in the first ultrasound assessment of the PI of UAs before the 30-min exercise training. The second ultrasound assessment of the PI of the left and right UAs took place after the 30-min exercise training. In the CG, each participant took part in the first and the second ultrasound assessment of the PI of the UAs, with each occurring 30 min apart. During the 30-min break, they did not practice any exercise and sat in a calm place. 

After one spontaneous menstrual cycle of the participants in the IG and CG, the degree of pain in the next menstrual period was assessed daily by all IG and CG participants during consecutive menstruation by completing the NRS [15,31] electronically, where 0 means no pain and 10 means unbearable pain.

The Borg scale is generally accepted as a tool for assessing perception of exercise intensity and has been used for many years as a self-report method for physical training participants [32]. The Borg scale scores were self-reported by the IG participants immediately after the exercise intervention, indicating how exhausting they found the exercise.

No data on the adverse effects of the practices were collected.

### 2.8. Statistical Methods

The chi-square test was used to measure the association between qualitative variables in the analysis. To compare the two groups using quantitative variables, an independent-samples test was used, the choice of which was conditional on normality being satisfied. Normality was tested using the Kolmogorov–Smirnov test, the result of which was used to decide whether normality could be assumed. The independent-samples *t*-test was used if the null hypothesis of normality was accepted, and the Mann–Whitney test was used if it was rejected. When the effect of several qualitative variables on quantity variables was investigated and an interaction term was included in the model, the ANCOVA was used. 

Exploratory data analysis was conducted on the Borg scale data, as this indicated that the scale scores weakly discriminated between subjects.

A power analysis was performed to demonstrate the sample size needed for specific levels of power based on Cohen’s d and partial eta-squared.

In each test, subjects missing any of the included data were excluded. The significance level was set at 0.05 for all tests and analyses were performed using IBM SPSS Statistics version 25.

### 2.9. Registry

The study was registered at clinicaltrials.gov: NCT04618172.

## 3. Results

### 3.1. Distribution of Demographic Data at Baseline

Demographic data at baseline in the IG and CG are shown in Table 1.

No significant difference was detected between the IG and the CG by using any demographic variable. The Kolmogorov–Smirnov normality test concluded that for the variables selected to confirm homogeneity, the two groups did not differ for any of the variables (age (year), weight (kg), Body Mass Index (BMI), duration of menstrual cycle, age at onset of menstruation (year), age at the first dysmenorrhea (year), number of deliveries). Since normality could not be assumed for any of the variables, the comparison was performed by using the non-parametric Mann–Whitney test.

### 3.2. Distribution of Participants in the IG and CG According to Menstrual Cycle Phase during Vaginal US

The distribution of participants in the IG and CG according to menstrual cycle phase during vaginal US is shown in Table 2.

There was no significant difference between IG and the CG regarding the phase of the menstrual cycle during vaginal US (χ^2^(2) = 2.653; *p* = 0.265).

### 3.3. Primary Outcome Measure

Examining the mean of the PI of UAs in the IG and the CG during the 1st and the 2nd measurement, a significant difference was found in the change in the measured value (Z = −2.545; *p* = 0.011). The IG showed a significantly higher increase in the mean of the PI (Median = 0.825) compared to the CG (Me = 0.130), as shown in Table 3.

#### Sample Size Calculations

PI UAs 1st US: Despite the medium effect size (Cohen’s d = 0.433), the relationship is not significant (*p* = 0.066); however, with a sample size of 84 (*n*(IG) = 44, *n*(CG) = 40), the difference would be significant, i.e., with no change in means, and variances in the significance value would be below 5%. If the power of the test was to be increased from the current 50% to 95%, a sample of 146 + 134 = 280 would be required for a significant result.

PI UAs 2nd US: Due to the relatively small effect size (Cohen’s d = 0.211), the difference between the two groups is not significant. If the power of the test was to be increased from 14% to 95%, a sample of 635 + 585 = 1220 would be needed for a significant result.

PI UAs D: The result is significant, and the effect size is medium-strong (Cohen’s d = 0.640) with a power of 76.8%, which if increased to 95%, would require a sample of 130 (*n*(IG) = 68, *n*(CG) = 62) to obtain a significant result at the 5% level. 

### 3.4. Secondary Outcome Measures

#### 3.4.1. The Results of the Average Level of Menstrual Pain Measured by Using the NRS in the IG and CG Are Presented in Table 4

When the change in the mean PI of UAs and the correlation between menstrual pain are examined, the fitted ANCOVA model does not show a significant correlation between the two variables. The model included participants’ age (F(1.66) = 0.165, *p* = 0.686), weight (F(1.66) = 0.283, *p* = 0.596) and BMI (F(1.66) = 1.316, *p* = 0.255) as control variables, none of which have a significant effect in the model. The outcome variable was the level of menstrual pain, as self-reported by using the NRS at the end of the study period, and the explanatory variables included PI UAs D (F(1.66) = 3.090, *p* = 0.083) in addition to the dummy variable group (F(1.66) = 0.872, *p* = 0.354) and the interaction between these two variables: Group × PI UAs D (F(1.66) = 0.940, *p* = 0.336). No significant effects were found, as shown in Table 5. 

**Table 4:** The average level of menstrual pain measured by using the NRS in the IC and CG.

Group	Mean	*n*	SD
IG	1.99505	38	1.641598
CG	2.588639	35	1.725807
Total	2.279648	73	1.697198

##### Sample Size Calculation

The relatively small effect size (η^2^ = 0.045) of the PI UAs D means that there is no significant difference between the means of the PI UAs 2nd US and PI UAs 1st US of the IG and CG. The power analysis demonstrated that, assuming no change in trends, a sample size of 143 would be required to confirm the significance of the present results.

#### 3.4.2. Adherence to the Intervention

In the IG, eight participants reported not performing the exercises twice weekly on a regular basis. These participants were subsequently assigned to the CG.

## 4. Discussion

The UAs are mainly responsible for the blood supply of the uterus [21]. The PI of UAs detected via ultrasound Doppler flowmetry measurement has gained more importance as it seems to more accurately describe the blood velocity waveform [22]. The PI values of the UAs are dependent on age and number of births; there were no significant differences between IG and CG members in these data in the demographic analysis [23]. 

The first hypothesis was confirmed. Examining the mean of the PI of UAs in the IG and the CG at the 1st and the 2nd US Doppler flowmetry measurement, a significant difference was found in the change in the measured value. The IG showed a significantly higher increase in the mean of the PI of UAs than the CG after exercise training. The elevated PI values of UAs demonstrated a reduced blood flow due to the circulatory redistribution in the participants of the IG. This change was driven by 30 min of exercise in the IG compared to the CG. The CG group also had 30 min between the two US measurements of PI of UAs, during which only rest was taken. In our study, IG participants completed the Borg scale to subjectively rate how physically demanding they found these exercises. According to the Borg scale, 91% of IG participants reported that they experienced mild to moderate exertion (Borg scale: 11–14; corresponding to 60–75% of the maximum target heart rate) [32].

During redistribution after exercise, blood flow increases not only in the skeletal muscle, but also in the respiratory and cardiac muscles, and modestly in the brain, depending on the magnitude and duration of the exercise load [33,34]. The blood flow responses of the uterus to exercise are very similar to those of visceral tissues. Thus, as part of the redistribution of blood flow away from non-muscle tissues such as the guts, kidneys and liver, blood flow is diverted from reproductive organs, including the uterus. To date, this research is the first in the literature to investigate the effect of exercise on UA PI values in PD individuals. The visceral redistribution of blood flow may be a component of oxygen conservation to meet the increased oxygen demand of the skeletal muscle during exercise and/or part of the reflex responses required to maintain arterial pressure during high-intensity exercise [35,36]. Our data are additional to the results of the abovementioned publications. These publications [33,34,35,36] were based on animal and human research and showed a reduction in blood flow in the uterus as a result of exercise. However, the change in the PI of UAs was not described in these papers.

Regarding our second hypothesis, there was no significant difference between the IG and CG regarding the PI of UAs and menstrual pain measured by using the Numeric Rating Scale (NRS). Menstrual pain did not differ significantly between the two groups during the short study period. The level of menstrual pain experienced by primary dysmenorrhea patients was independent of the level of blood circulation regarding the PI of the UAs. However, in the current literature, systematic reviews and meta-analyses [17,37] mentioned in the introduction section, regular exercise interventions are recommended for pain relief from PD. In our other research article, under peer-review, we studied the relationship between a longer period of exercise and level of menstrual pain in patients with PD. The pain-relieving effect of regular exercise on PD manifests in other ways, not through the change in the PI of UAs. The most important pathophysiological factor in the development of PD is the increased level of prostaglandins (mainly PGE2 and PGF2α) in the menstrual blood [3,38]. Pro-inflammatory cytokines such as TNFα, IL-6 also play a role in the development of PD [39,40,41]. Previous research has shown that increasing progesterone levels through exercise can lead to a reduction in the production of prostaglandins and pro-inflammatory cytokines and, thus, a reduction in pain [19,42]. In the corresponding literature, physical exercises are also known to increase the level of endorphins and endocannabinoids in the blood [43,44,45]. Short-term exercise reduces cortisol production and has a non-specific analgesic effect [46].

However, further studies are needed to confirm our findings regarding a longer research period on the association between regular (twice a week or more) mild-to-moderate exercise and the PI of UA in the IG and the healthy CG. Another question is whether there is a compensatory postexercise vasodilatation [47] in the PI of UAs during recovery from exercise intervention in the IG, changing a decreased blood flow to an increased blood flow. Another research topic is how exercise changes the blood flow in the ovarian arteries, which is also involved, to a lesser extent, in the uterine blood supply in the context of anastomosis.

### Strengths and Limitations

A strength of the study is the assessment of the significant effects of exercise training on the change in the PI of the UAs in individuals with PD for the first time. Another strength is the flexible and realistic research, not only with adolescents but also women of all ages in the reproductive life stage who volunteered to participate in the study. Most research on PD only focuses on teenagers and young women.

The limitations of our study were the relatively small number of patients, the duration of each cycle assessment, short detection period of the study and the prospective observational study design. Self-selection was another limitation because it removed all the benefits of randomisation, such as balancing confounding factors.

## 5. Conclusions

Our study is the first to document the significant effects of mild-to-moderate exercise on changes in the PI in UAs in individuals with PD. The IG had a significantly elevated PI in UAs compared to the CG after 30 min of exercise. This demonstrated a reduced blood flow due to circulatory redistribution in the participants in the IG. The level of menstrual pain in PD patients is independent of the level of blood circulation regarding the PI of the UAs. 

Due to the limitations of our study, further research is needed to verify these results.

Due to the relatively small number of patients, the short study period and the prospective observational study design, our results need to be confirmed in larger clinical trials. 

## Figures and Tables

**Table 1:** Distribution of demographic data between the two groups at baseline.

							K-S Test ^a^		M-W Test ^b^	
Variables	Group	*n*	Mean	Me *	SD **	D	df	*p*	Z	*p*
Age distribution (year)	CG	35	33.140	33.0	6.236	0.105	35	0.200	−0.216	0.829
	IG	38	33.290	33.50	6.093	0.149	38	0.033		
Weight (kg)	CG	35	65.460	63.00	13.393	0.14	35	0.081	−1.724	0.085
	IG	38	59.950	56.00	10.608	0.171	38	0.006		
Body mass index (BMI)	CG	35	23.452	22.31	4.947	0.164	35	0.018	−1.099	0.272
	IG	38	21.906	21.41	3.714	0.114	38	0.200		
Duration of the menstrual cycle (day)	CG	35	30.570	28.00	9.391	0.256	35	<0.001	−1.425	0.154
	IG	38	28.050	28.00	3.170	0.138	38	0.065		
Age at onset of menstuation (year)	CG	32	12.720	12.50	1.442	0.191	32	0.004	−1.276	0.202
	IG	33	13.150	13.00	1.349	0.211	33	0.001		
Age at the first dysmenorrhea (year)	CG	31	15.580	14.00	5.458	0.204	31	0.002	−0.959	0.338
	IG	27	16.040	15.00	4.174	0.244	27	<0.001		
Number of deliveries	CG	33	0.150	0.00	0.619	0.483	32	<0.001	−1.426	0.154
	IG	32	0.250	0.00	0.568	0.536	33	<0.001		

* Median; ** Standard deviation; ^a^ Kolmogorov–Smirnov test; ^b^ Mann–Whitney test.

**Table 2:** The distribution of participants in the IG and CG according to menstrual cycle phase during vaginal US.

Time of US *	IG	CG	Total
follicular phase	13	10	23
ovulation phase	10	5	15
luteal phase	15	20	35
Total	38	35	73

* ultrasound *p* = 0.265.

**Table 3:** Mean PI in UAs during the 1st and 2nd US measurement of the IG and the CG.

						K-S Test ^a^			LTfEoV ^b^		*t*-Test		M-W Test ^c^	
	Group	*n*	Mean	SD *	Me **	D	df	*p*	F	*p*	*t*	*p*	Z	*p*
PI UAs 1st US	IG	38	2.571	0.969	2.480	0.097	38	0.200	2.547	0.115	−1.864	0.066	NA	NA
	CG	35	3.088	1.379	2.795	0.125	35	0.180						
PI UAs 2nd US	IG	38	3.476	1.989	3.043	0.217	38	<0.001					−0.392	0.695
	CG	35	3.118	1.328	2.860	0.145	35	0.060						
PI UAs D	IG	38	0.905	1.651	0.825	0.215	38	<0.001					−2.545	0.011
	CG	35	0.030	1.006	0.130	0.123	35	0.200						

* Standard deviation; ** Median; ^a^ Kolmogorov–Smirnov test; ^b^ Levene’s Test for Equality of Variances; ^c^ Mann–Whitney test; PI UAs 1st US: Mean PI in UAs during the 1st US measurement of the IG and the CG; PI UAs 2nd US: Mean PI in UAs during the 2nd US measurement of the IG and the CG; PI UAs D: Difference between the means of the PI UAs 2nd US and PI UAs 1st US.

**Table 5:** Statistics of the ANCOVA model fitted to the level of menstrual pain at the end of the study (with the variable of group membership as an explanatory and moderating variable).

Source	Type III Sum of Squares	df	Mean Square	F	*p*	Partial Eta Squared
Corrected Model	27.269	6	4.545	1.665	0.143	0.131
Intercept	1.473	1	1.473	0.540	0.465	0.008
Group ^a^	2.380	1	2.380	0.872	0.354	0.013
PI UAs D	8.433	1	8.433	3.090	0.083	0.045
Group × PI UAs D ^b^	2.564	1	2.564	0.940	0.336	0.014
Age (years)	0.451	1	0.451	0.165	0.686	0.002
Weight (kg)	0.773	1	0.773	0.283	0.596	0.004
BMI	3.591	1	3.591	1.316	0.255	0.020
Error	180.125	66	2.729			
Total	586.761	73				
Corrected Total	207.395	72				

^a^ IG and CG altogether; ^b^ PI UAs D: Difference between the means of the PI UAs 2nd US and PI UAs 1st US.

## Data Availability

The data used to support the findings of this study are available from the corresponding author upon request.
